# Supplementary material for: Lactate biosensors for spectrally and spatially multiplexed fluorescence imaging
Source: Nat Commun. 2023 Oct 27;14:6598. doi: 10.1038/s41467-023-42230-5 (PMC10611801; doi:10.1038/s41467-023-42230-5)
Supplement: Supplementary file 1 — Supplementary information [file 41467_2023_42230_MOESM1_ESM.pdf]

Supplementary information

Lactate biosensors
for spectrally and spatially multiplexed fluorescence imaging

Figure	
Supplementary Figure 1	eLACCO0.9 exhibits a faster response than eLACCO1.
Supplementary Figure 2	Development of affinity-tuned eLACCO2.1.
Supplementary Figure 3	Sequence alignment of TTHA0766, cpGFP, eLACCO1.1, eLACCO2.1, and deLACCO1.
Supplementary Figure 4	*In vitro* characterization of deLACCO1 and R-diLACCO1.
Supplementary Figure 5	Lineage of eLACCO and R-iLACCO variants.
Supplementary Figure 6	*In vitro* characterization of eLACCO2.1.
Supplementary Figure 7	Construction of the R-iLACCO prototype.
Supplementary Figure 8	Sequence alignment of LldR, cpmApple, R-iLACCO1, R-iLACCO1.1, R-iLACCO1.2, and R-diLACCO1.
Supplementary Figure 9	Development of R-iLACCO variants with lower L-lactate affinity.
Supplementary Figure 10	*In vitro* characterization of R-iLACCO1.
Supplementary Figure 11	*In vitro* characterization of R-iLACCO1.1 and R- [illegible]
Supplementary Figure 12	Fluorescence imaging of the other currently-available intracellular L-lactate biosensors.
Supplementary Figure 13	Inhibition of L-lactate transporter perturbs intracellular L-lactate and pH dynamics.
Supplementary Figure 14	Imaging of R-iLACCO variants in various tissues.
Supplementary Figure 15	Fluorescence imaging of eLACCO2.1 in extracellular Ca^{2+} concentration range from 1 to 2 mM.
Supplementary Figure 16	Temperature-dependent lactate response of R-iLACCO variants.
**Table**	
Supplementary Table 1	Biochemical parameters of eLACCO variants.
Supplementary Table 2	Biochemical parameters of R-iLACCO variants.
**Note**	
Supplementary Note 1	*In vitro* characterization of eLACCO2.1.
Supplementary Note 2	*In vitro* characterization of R-iLACCO1.
Supplementary Note 3	Importance of leader and anchor optimization for extracellular biosensors
**Reference**	
Supplementary References	

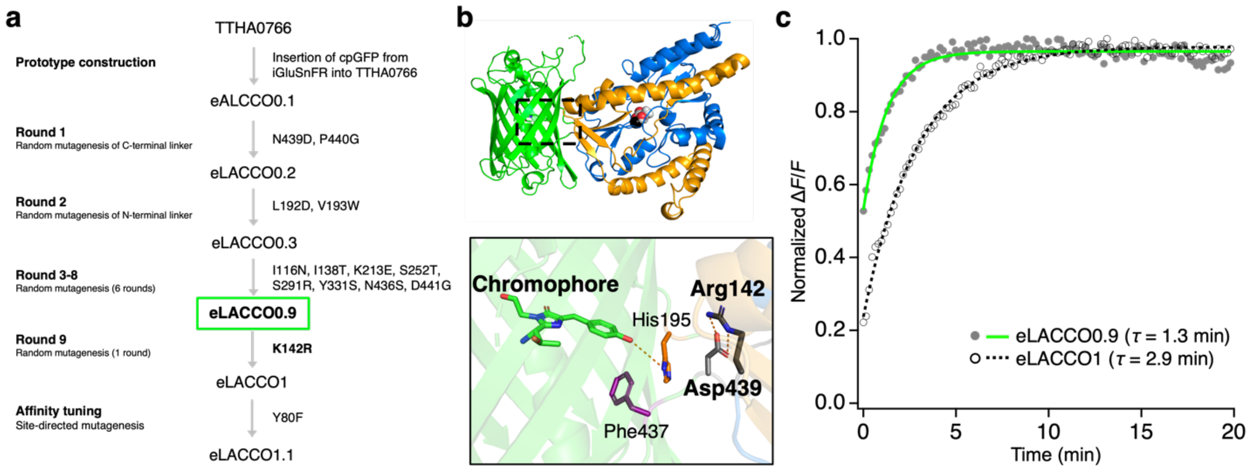

Supplementary Figure 1. eLACCO0.9 exhibits a faster response than eLACCO1.

(**a**) Lineage of previously reported eLACCO variants (ref. [1]). The final round (round 9) of directed evolution via random mutagenesis introduced the Lys142Arg mutation into eLACCO0.9 to produce eLACCO1. (**b**) Crystal structure (PDB ID: 7E9Y) of eLACCO1 in L-lactate bound state. Inset represents a zoom-in view around the chromophore. Arg142 forms a salt bridge with Asp439. (**c**) Time courses of fluorescence response of eLACCO0.9 and eLACCO1. L-Lactate (final concentration of 10 mM) was rapidly mixed at t = 0 (dead time ~ 10 s) with the crude protein extracts. Curve fitting was performed using $\Delta F/F = A + B \cdot e^{-t/\tau}$, where τ is a time constant.

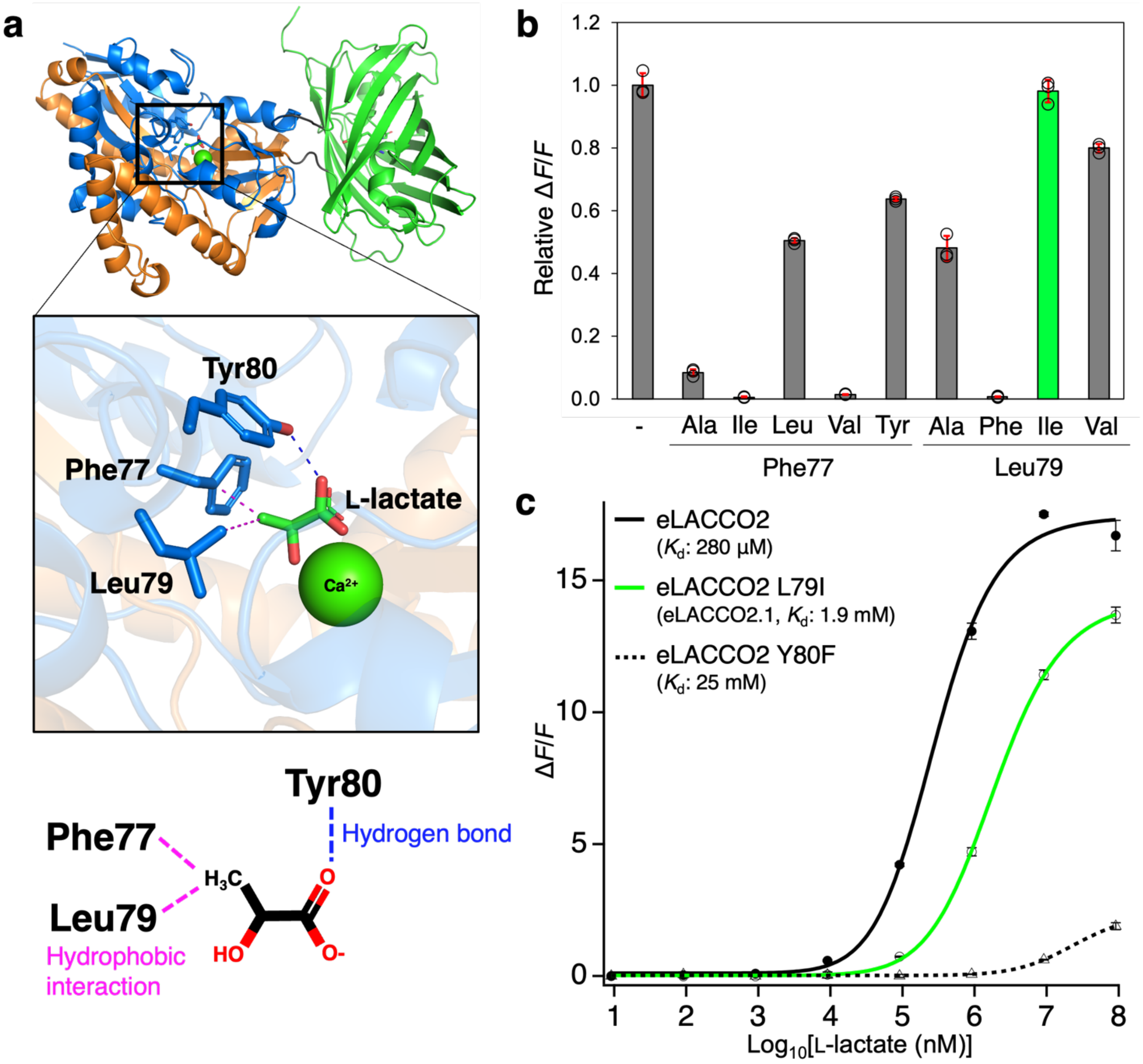

Supplementary Figure 2. Development of affinity-tuned eLACCO2.1.

(**a**) Crystal structure of eLACCO1 (PDB ID: 7E9Y)[1] and zoom-in view of the L-lactate binding pocket. The phenol group of the Tyr80 side chain forms a hydrogen bond to the carboxylate group of L-lactate. Hydrophobic side chains of Phe77 and Leu79 interact with a methyl group of L-lactate. (**b**) Relative Δ*F*/*F* of a range of eLACCO2 variants. $n = 3$ experimental triplicates (mean ± s.d.). (**c**) Dose-response curves of eLACCO2, eLACCO2 Leu79Ile (eLACCO2.1), and eLACCO2 Tyr80Phe for L-lactate. $n = 3$ experimental triplicates (mean ± s.d.).

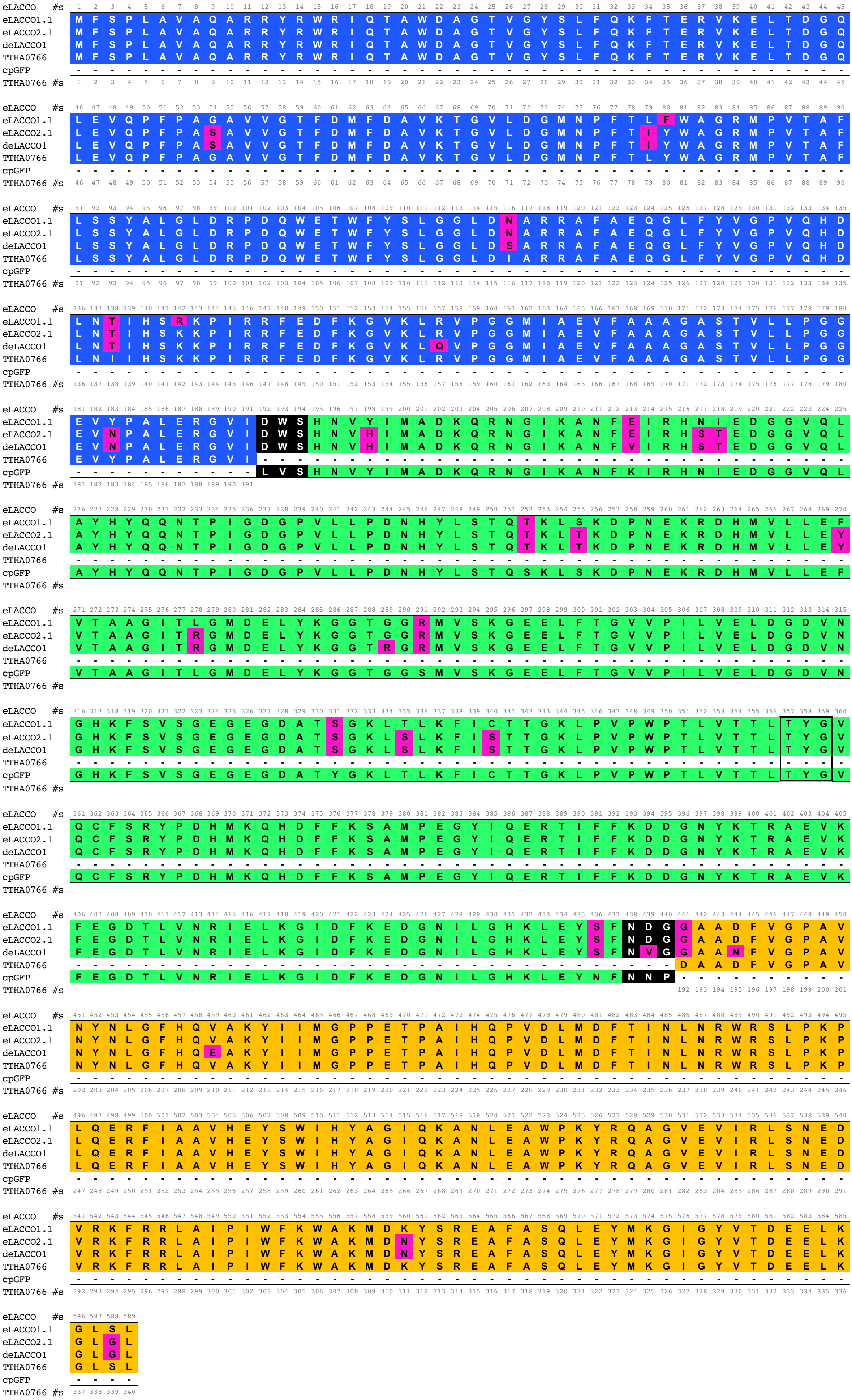
eLACCO
eLACCO1.1
eLACCO2.1
deLACCO1
TTHA0766
cpGFP
TTHA0766 #s

Supplementary Figure 3. Sequence alignment of TTHA0766, cpGFP, eLACCO1.1, eLACCO2.1, and deLACCO1.

Mutations in eLACCO variants, relative to TTHA0766 and cpGFP, are highlighted in magenta. The chromophore-forming residues are surrounded by a double line.

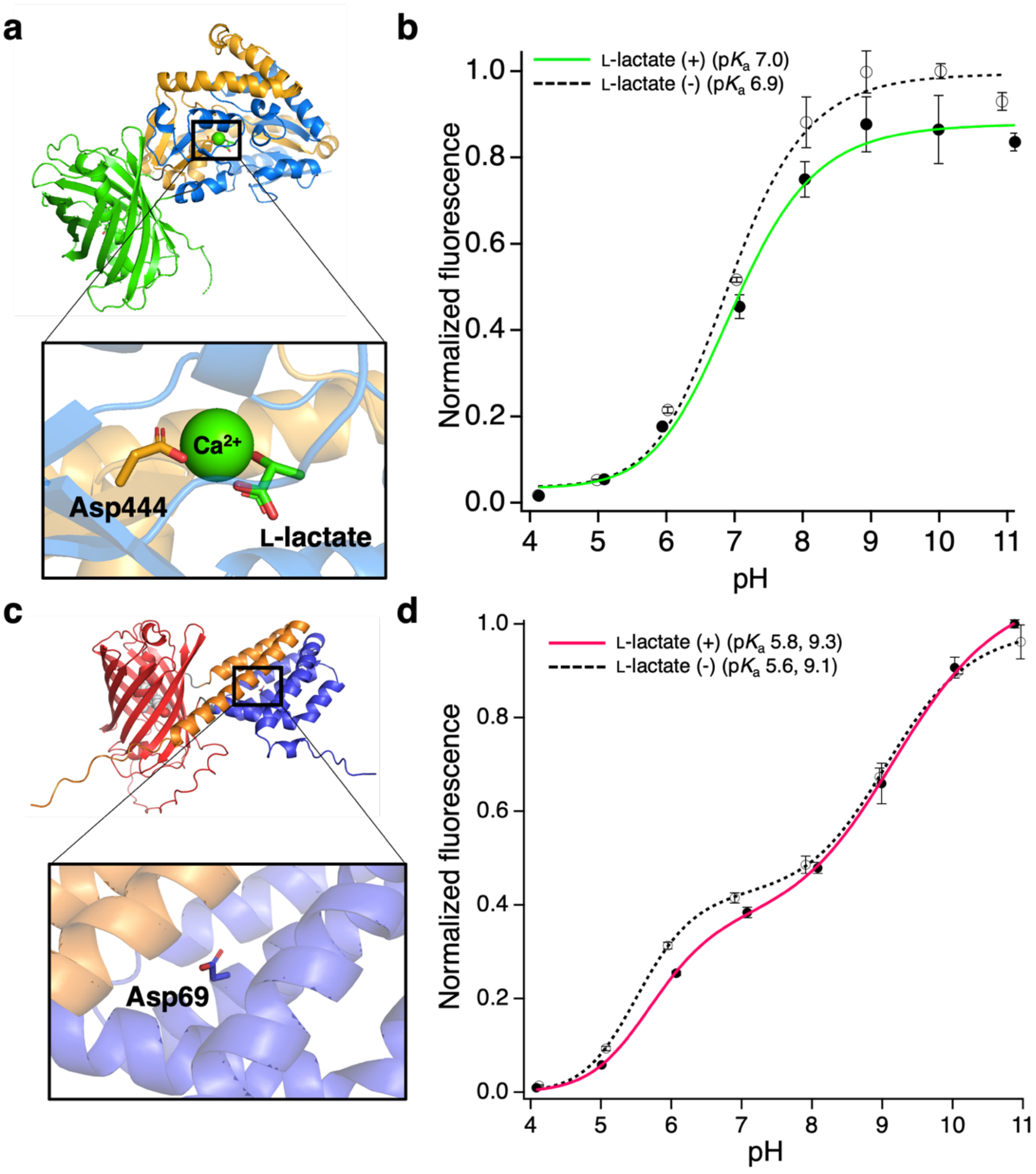

Supplementary Figure 4. *In vitro* characterization of deLACCO1 and R-diLACCO1.

(**a**) Crystal structure of eLACCO1 (ref. [1]) and zoom-in view of the Ca^{2+} binding pocket. Carboxyl group of Asp444 side chain coordinates to Ca^{2+}. (**b**) pH titration curves of purified deLACCO1 in the presence (100 mM) and absence of L-lactate. $n = 3$ experimental triplicates (mean ± s.d.). (**c**) AlphaFold model of R-iLACCO1 and zoom-in view of the putative L-lactate binding pocket. (**d**) pH titration curves of purified R-diLACCO1 in the presence (100 mM) and absence of L-lactate. $n = 3$ experimental triplicates (mean ± s.d.).

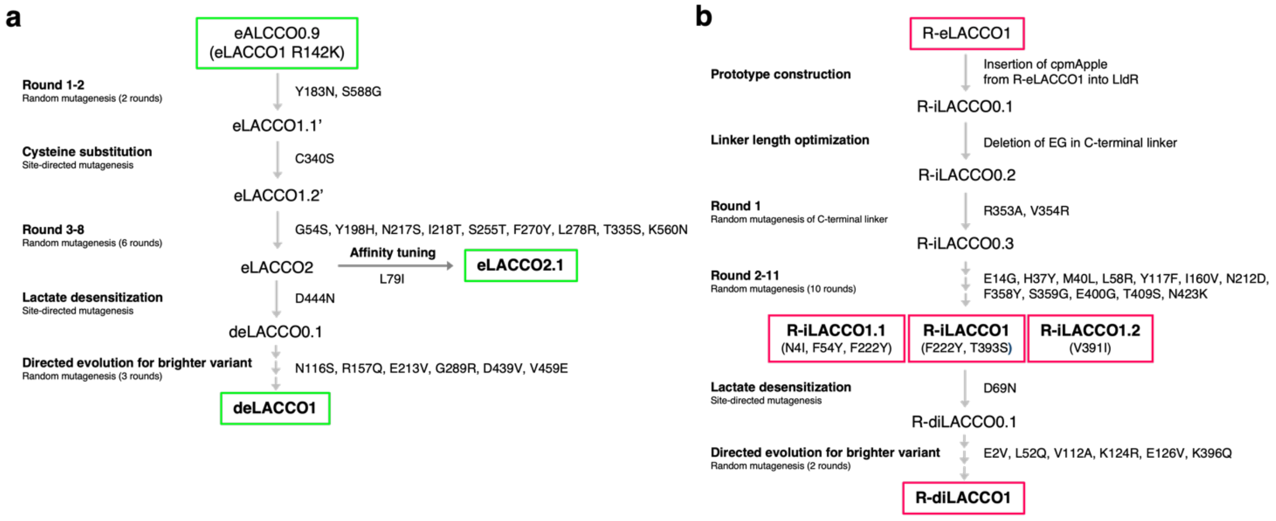

Supplementary Figure 5. Lineage of eLACCO and R-iLACCO variants.

(**a**) Lineage of eLACCO variants. (**b**) Lineage of R-iLACCO variants.

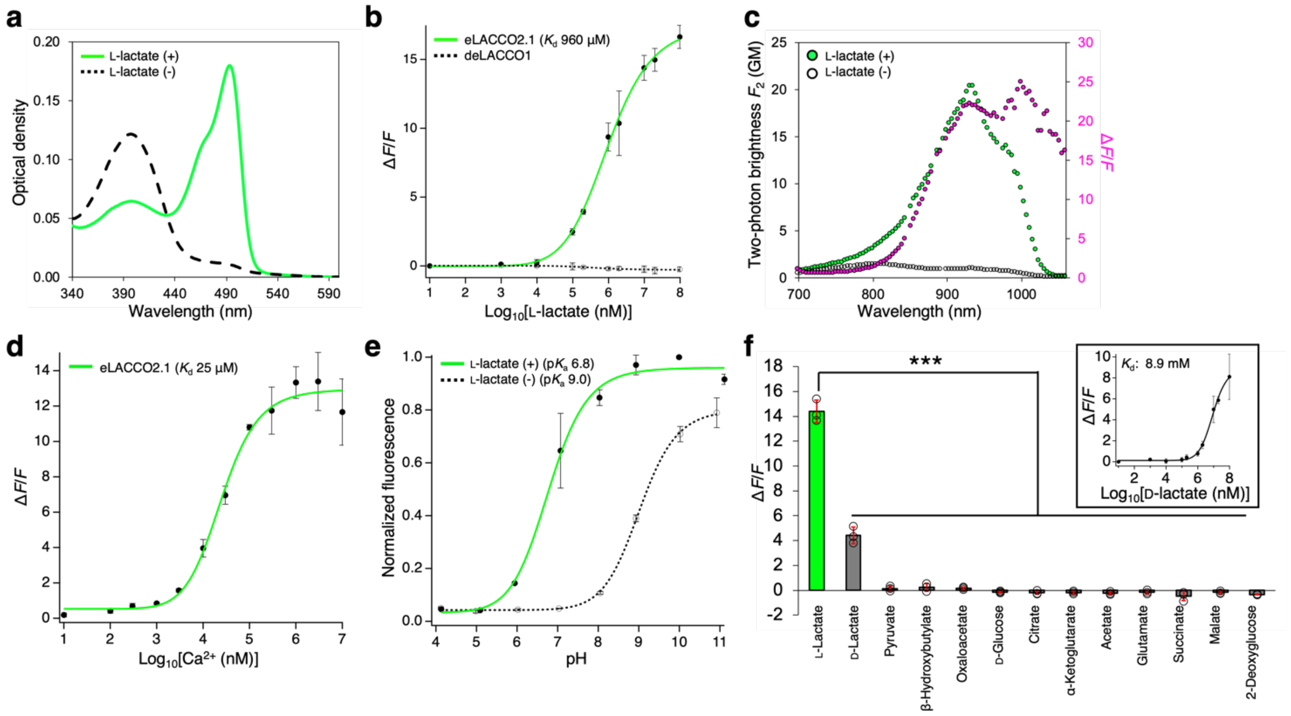

Supplementary Figure 6. *In vitro* characterization of eLACCO2.1.

(**a**) Absorbance spectra of eLACCO2.1 in the presence (10 mM) and absence of L-lactate. (**b**) Dose-response curves of eLACCO2.1 and deLACCO1 for L-lactate. *n* = 3 experimental triplicates (mean ± s.d.). (**c**) Two-photon excitation spectra of eLACCO2.1 in the presence (10 mM) and absence of L-lactate. Δ*F*/*F* is represented in the magenta plots. GM, Goeppert-Mayer units. (**d**) Dose-response curve of eLACCO2.1 as a function of Ca^{2+} in the presence (100 mM) and absence of L-lactate. *n* = 3 experimental triplicates (mean ± s.d.). (**e**) pH titration curves of eLACCO2.1 in the presence (100 mM) and absence of L-lactate. *n* = 3 experimental triplicates (mean ± s.d.). (**f**) Pharmacological specificity of eLACCO2.1. Concentration of each metabolite is 10 mM. Inset is a dose-response curve of eLACCO2.1 for D-lactate. *n* = 3 experimental triplicates (mean ± s.d.). Statistical analysis was performed using one-way analysis of variance (ANOVA) with the Dunnett's post hoc tests. ***$p < 0.0001$.

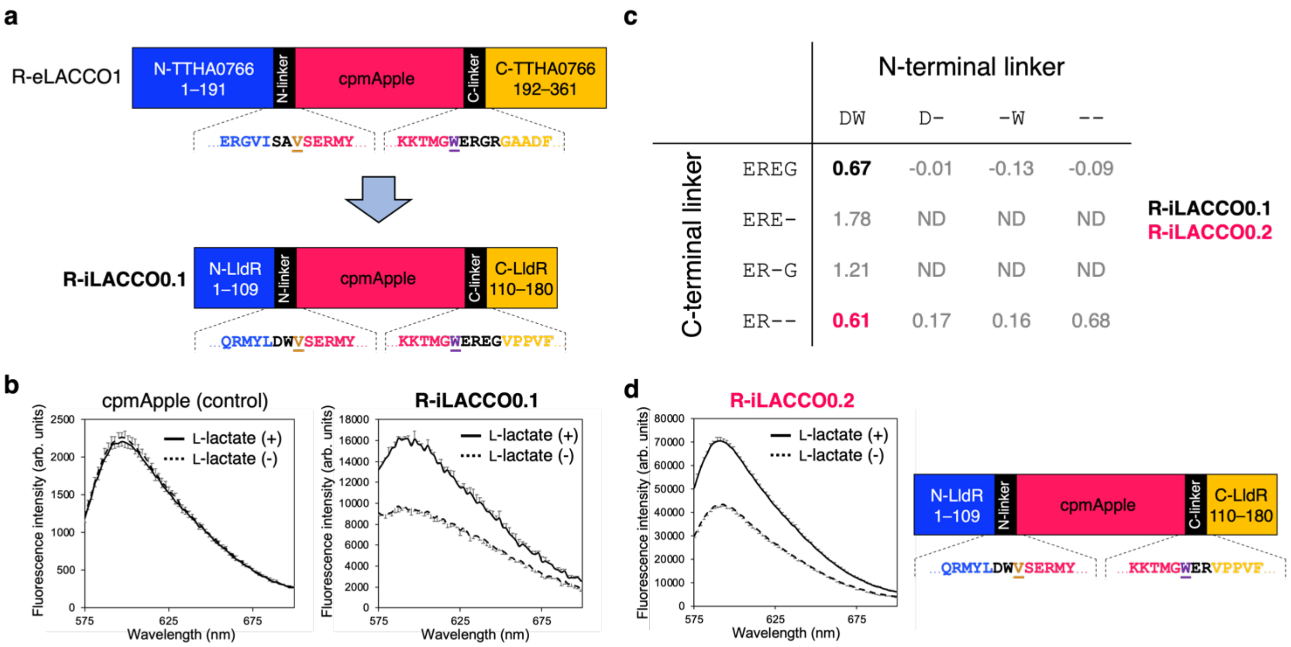

Supplementary Figure 7. Construction of the R-iLACCO prototype.

(**a**) Schematic illustration of the biosensor prototype construction based on R-eLACCO1 (ref. [2]). Linker regions are shown in black and the two "gate post" residues[3] in cpmApple are highlighted in dark orange and purple. (**b**) Emission spectra of R-iLACCO0.1 and cpmApple in the presence (10 mM) and absence of L-lactate. Error bars represent standard deviation of triplicates. (**c**) Summary of $\Delta F/F$ of R-iLACCO variants with different linker length. ND, not determined. (**d**) Emission spectra of R-iLACCO0.2 in the presence (10 mM) and absence of L-lactate. Right figure shows a schematic representation of the primary structure of R-iLACCO0.2. Error bars represent standard deviation of triplicates.

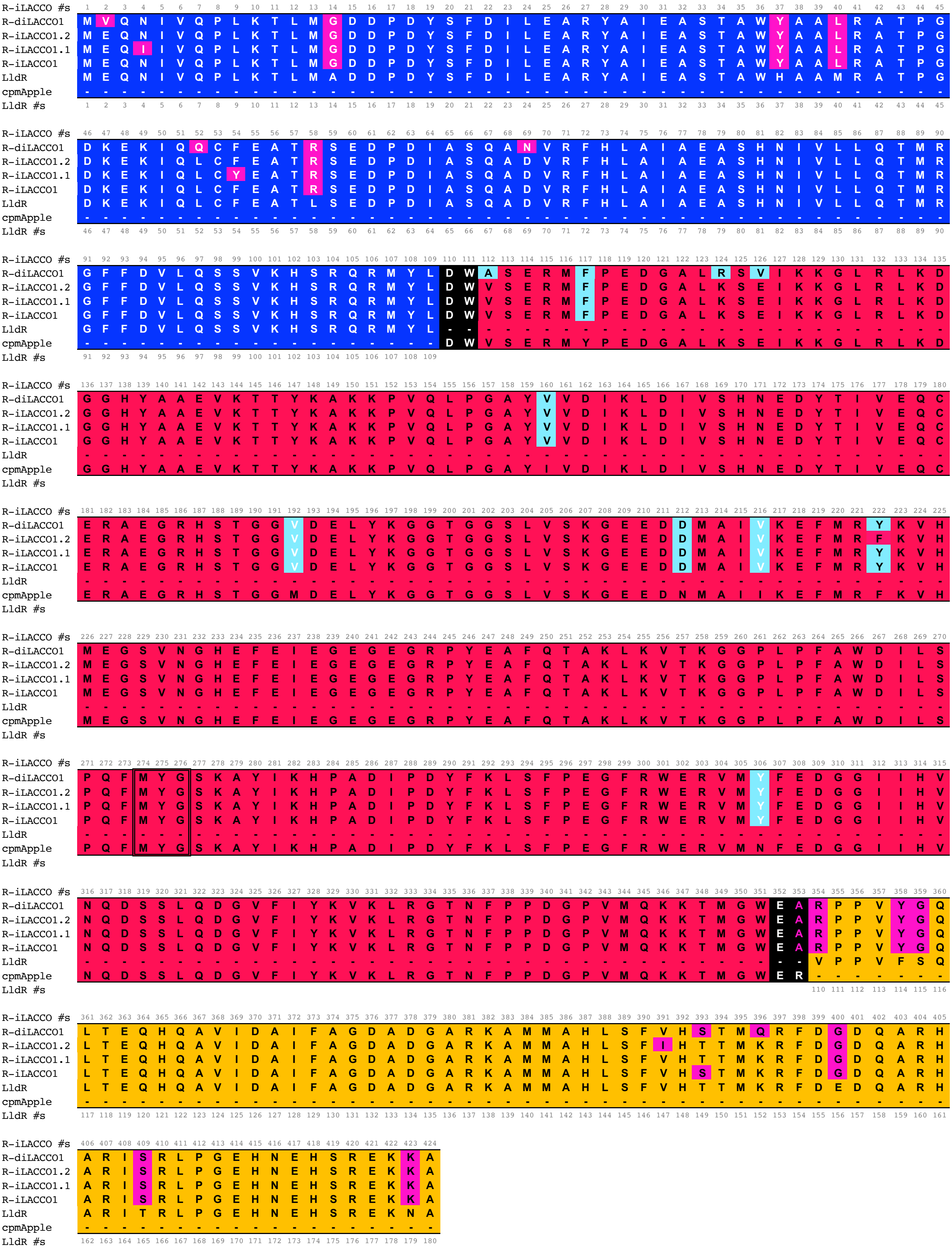
R-iLACCO #s 1 … 45
R-diLACCO1 MVQNIVQPLKTLMGDDDPDYSFDILEARYAIEASTAWYAALRATPG
R-iLACCO1.2 MEQNIVQPLKTLMGDDDPDYSFDILEARYAIEASTAWYAALRATPG
R-iLACCO1.1 MEQIIVQPLKTLMGDDDPDYSFDILEARYAIEASTAWYAALRATPG
R-iLACCO1 MEQNIVQPLKTLMGDDDPDYSFDILEARYAIEASTAWYAALRATPG
LldR MEQNIVQPLKTLMADDDPDYSFDILEARYAIEASTAWHAAMRATPG
cpmApple ---
LldR #s 1 … 45
R-iLACCO #s 46 … 90
R-diLACCO1 DKEKIQQCFEATRSEDPDIASQANVRFHLAIAEASHNIVLLQTMR
R-iLACCO1.2 DKEKIQLCFEATRSEDPDIASQADVRFHLAIAEASHNIVLLQTMR
R-iLACCO1.1 DKEKIQLCYEATRSEDPDIASQADVRFHLAIAEASHNIVLLQTMR
R-iLACCO1 DKEKIQLCFEATRSEDPDIASQADVRFHLAIAEASHNIVLLQTMR
LldR DKEKIQLCFEATLSEDPDIASQADVRFHLAIAEASHNIVLLQTMR
cpmApple ---
LldR #s 46 … 90
R-iLACCO #s 91 … 135
R-diLACCO1 GFFDVLQSSVKHSRQRMYLDWASERMFPEDGALRSVIKKGLRLKD
R-iLACCO1.2 GFFDVLQSSVKHSRQRMYLDWVSERMFPEDGALKSEIKKGLRLKD
R-iLACCO1.1 GFFDVLQSSVKHSRQRMYLDWVSERMFPEDGALKSEIKKGLRLKD
R-iLACCO1 GFFDVLQSSVKHSRQRMYLDWVSERMFPEDGALKSEIKKGLRLKD
LldR GFFDVLQSSVKHSRQRMYL--------------------------
cpmApple -------------------DWVSERMYPEDGALKSEIKKGLRLKD
LldR #s 91 … 109
R-iLACCO #s 136 … 180
R-diLACCO1 GGHYAAEVKTTYKAKKPVQLPGAYVVDIKLDIVSHNEDYTIVEQC
R-iLACCO1.2 GGHYAAEVKTTYKAKKPVQLPGAYVVDIKLDIVSHNEDYTIVEQC
R-iLACCO1.1 GGHYAAEVKTTYKAKKPVQLPGAYVVDIKLDIVSHNEDYTIVEQC
R-iLACCO1 GGHYAAEVKTTYKAKKPVQLPGAYVVDIKLDIVSHNEDYTIVEQC
LldR ---
cpmApple GGHYAAEVKTTYKAKKPVQLPGAYIVDIKLDIVSHNEDYTIVEQC
LldR #s
R-iLACCO #s 181 … 225
R-diLACCO1 ERAEGRHSTGGVDELYKGGTGGSLVSKGEEDDMAIVKEFMRYKVH
R-iLACCO1.2 ERAEGRHSTGGVDELYKGGTGGSLVSKGEEDDMAIVKEFMRFKVH
R-iLACCO1.1 ERAEGRHSTGGVDELYKGGTGGSLVSKGEEDDMAIVKEFMRYKVH
R-iLACCO1 ERAEGRHSTGGVDELYKGGTGGSLVSKGEEDDMAIVKEFMRYKVH
LldR ---
cpmApple ERAEGRHSTGGMDELYKGGTGGSLVSKGEEDNMAIIKEFMRFKVH
LldR #s
R-iLACCO #s 226 … 270
R-diLACCO1 MEGSVNGHEFEIEGEGEGRPYEAFQTAKLKVTKGGPLPFAWDILS
R-iLACCO1.2 MEGSVNGHEFEIEGEGEGRPYEAFQTAKLKVTKGGPLPFAWDILS
R-iLACCO1.1 MEGSVNGHEFEIEGEGEGRPYEAFQTAKLKVTKGGPLPFAWDILS
R-iLACCO1 MEGSVNGHEFEIEGEGEGRPYEAFQTAKLKVTKGGPLPFAWDILS
LldR ---
cpmApple MEGSVNGHEFEIEGEGEGRPYEAFQTAKLKVTKGGPLPFAWDILS
LldR #s
R-iLACCO #s 271 … 315
R-diLACCO1 PQFMYGSKAYIKHPADIPDYFKLSFPEGFRWERVMYFEDGGIIHV
R-iLACCO1.2 PQFMYGSKAYIKHPADIPDYFKLSFPEGFRWERVMYFEDGGIIHV
R-iLACCO1.1 PQFMYGSKAYIKHPADIPDYFKLSFPEGFRWERVMYFEDGGIIHV
R-iLACCO1 PQFMYGSKAYIKHPADIPDYFKLSFPEGFRWERVMYFEDGGIIHV
LldR ---
cpmApple PQFMYGSKAYIKHPADIPDYFKLSFPEGFRWERVMNFEDGGIIHV
LldR #s
R-iLACCO #s 316 … 360
R-diLACCO1 NQDSSLQDGVFIYKVKLRGTNFPPDGPVMQKKTMGWEARPPVYGQ
R-iLACCO1.2 NQDSSLQDGVFIYKVKLRGTNFPPDGPVMQKKTMGWEARPPVYGQ
R-iLACCO1.1 NQDSSLQDGVFIYKVKLRGTNFPPDGPVMQKKTMGWEARPPVYGQ
R-iLACCO1 NQDSSLQDGVFIYKVKLRGTNFPPDGPVMQKKTMGWEARPPVYGQ
LldR ---------------------------------------VPPVFSQ
cpmApple NQDSSLQDGVFIYKVKLRGTNFPPDGPVMQKKTMGWER-------
LldR #s 110 … 116
R-iLACCO #s 361 … 405
R-diLACCO1 LTEQHQAVIDAIFAGDADGARKAMMAHLSFVHSTMQRFDGDQARH
R-iLACCO1.2 LTEQHQAVIDAIFAGDADGARKAMMAHLSFIHTTMKRFDGDQARH
R-iLACCO1.1 LTEQHQAVIDAIFAGDADGARKAMMAHLSFVHTTMKRFDGDQARH
R-iLACCO1 LTEQHQAVIDAIFAGDADGARKAMMAHLSFVHSTMKRFDGDQARH
LldR LTEQHQAVIDAIFAGDADGARKAMMAHLSFVHTTMKRFDEDQARH
cpmApple ---
LldR #s 117 … 161
R-iLACCO #s 406 … 424
R-diLACCO1 ARISRLPGEHNEHSREKKA
R-iLACCO1.2 ARISRLPGEHNEHSREKKA
R-iLACCO1.1 ARISRLPGEHNEHSREKKA
R-iLACCO1 ARISRLPGEHNEHSREKKA
LldR ARITRLPGEHNEHSREKNA
cpmApple -------------------
LldR #s 162 … 180

Supplementary Figure 8. Sequence alignment of LldR, cpmApple, R-iLACCO1, R-iLACCO1.1, R-iLACCO1.2, and R-diLACCO1.

Mutations in R-iLACCO variants, relative to LldR and cpmApple, are highlighted in magenta and light blue, respectively. White residues in light blue represent mutations in cpmApple derived from R-eLACCO1 (ref. [2]). The chromophore-forming residues are surrounded by a double line.

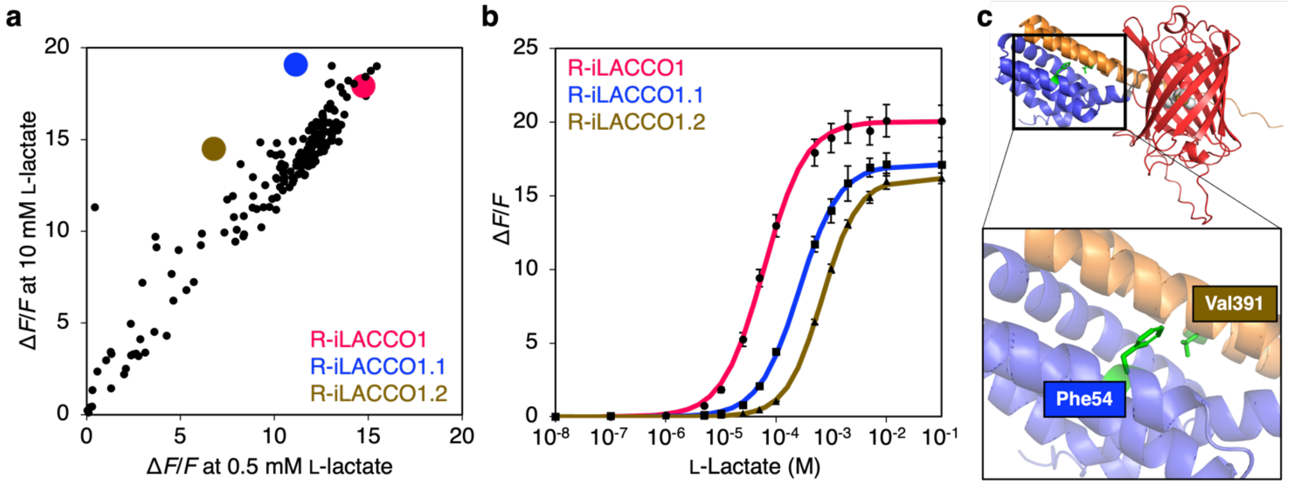

Supplementary Figure 9. Development of R-iLACCO variants with lower L-lactate affinity.

(**a**) Scatter plot representing Δ*F*/*F* of all proteins tested in the final round of directed evolution for R-iLACCO. Δ*F*/*F* for each protein was measured at both 0.5 mM and 10 mM L-lactate. Red, blue, and brown filled circles indicate R-iLACCO1, R-iLACCO1.1, and R-iLACCO1.2, respectively. (**b**) Dose-response curves of R-iLACCO1, R-iLACCO1.1 and R-iLACCO1.2 for L-lactate. *n* = 3 experimental triplicates (mean ± s.d.). (**c**) AlphaFold model of R-iLACCO1 and zoom-in view of the putative L-lactate binding pocket. Note that, relative to R-iLACCO1, the low affinity variants R-iLACCO1.1 and R-iLACCO1.2 have F54Y and V391I, respectively, both of which are toward the putative L-lactate binding pocket.

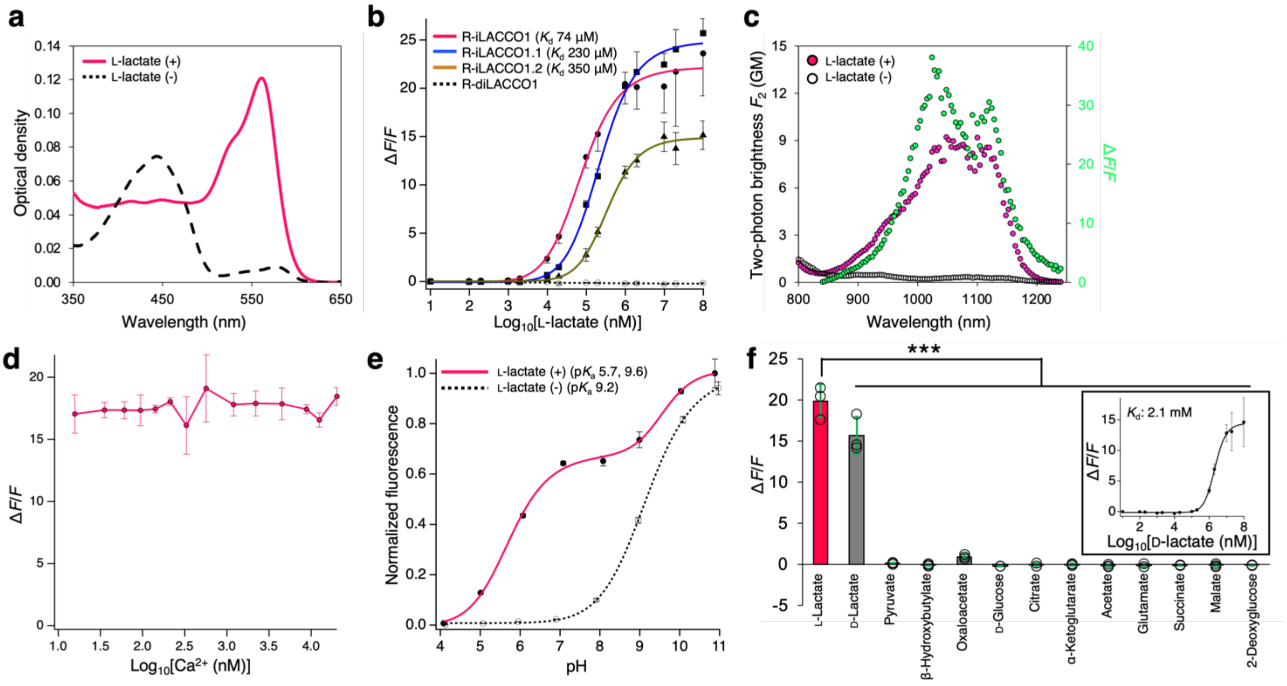

Supplementary Figure 10. *In vitro* characterization of R-iLACCO1.

(**a**) Absorbance spectra of R-iLACCO1 in the presence (10 mM) and absence of L-lactate. (**b**) Dose-response curves of R-iLACCO1 and its affinity variants for L-lactate. $n = 3$ experimental triplicates (mean ± s.d.). (**c**) Two-photon excitation spectra of R-iLACCO1 in the presence (10 mM) and absence of L-lactate. $\Delta F/F$ is represented in the green plots. GM, Goeppert-Mayer units. (**d**) $\Delta F/F$ plot of R-iLACCO1 as a function of Ca^{2+} in treatment with 100 mM L-lactate. $n = 3$ experimental triplicates (mean ± s.d.). (**e**) pH titration curves of R-iLACCO1 in the presence (100 mM) and absence of L-lactate. $n = 3$ experimental triplicates (mean ± s.d.). (**f**) Pharmacological specificity of R-iLACCO1. Concentration of each metabolite is 10 mM. Inset is a dose-response curve of R-iLACCO1 for D-lactate. $n = 3$ experimental triplicates (mean ± s.d.). Statistical analysis was performed using one-way ANOVA with the Dunnett's post hoc tests. ***$p < 0.0001$.

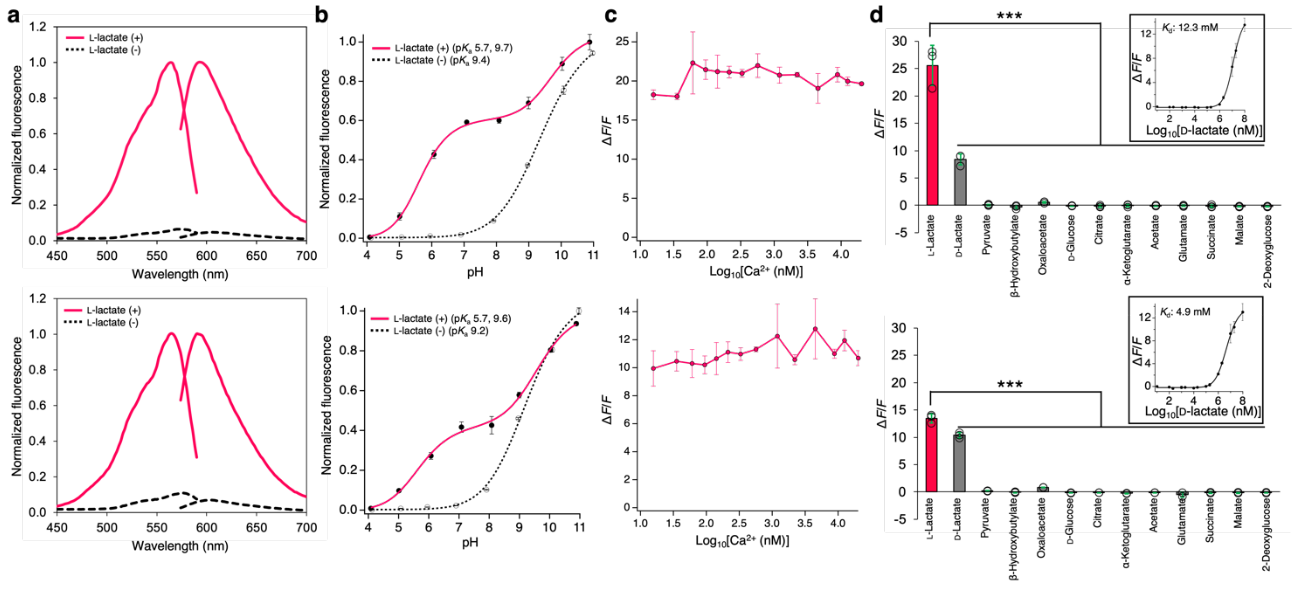

Supplementary Figure 11. ***In vitro*** **characterization of R-iLACCO1.1 and R-iLACCO1.2.** (**a**) Excitation and emission spectra of R-iLACCO1.1 (upper) and R-iLACCO1.2 (bottom) in the presence (10 mM) and absence of L-lactate. (**b**) pH titration curves of R-iLACCO1.1 (upper) and R-iLACCO1.2 (bottom) in the presence (100 mM) and absence of L-lactate. n = 3 experimental triplicates (mean ± s.d.). (**c**) $\Delta F/F$ plot of R-iLACCO1.1 (upper) and R-iLACCO1.2 (bottom) as a function of Ca^{2+} in treatment with 100 mM L-lactate. n = 3 experimental triplicates (mean ± s.d.). (**d**) Pharmacological specificity of R-iLACCO1.1 (upper) and R-iLACCO1.2 (bottom). Concentration of each metabolite is 10 mM. Insets are dose-response curves for D-lactate. n = 3 experimental triplicates (mean ± s.d.). One data point of citrate, α-ketoglutarate, glutamate, succinate, malate, and 2-deoxyglucose for R-iLACCO1.2 was omitted from the bar graph in **d** because it was an outlier. Statistical analyses were performed using one-way ANOVA with the Dunnett's post hoc tests. ***$p < 0.0001$.

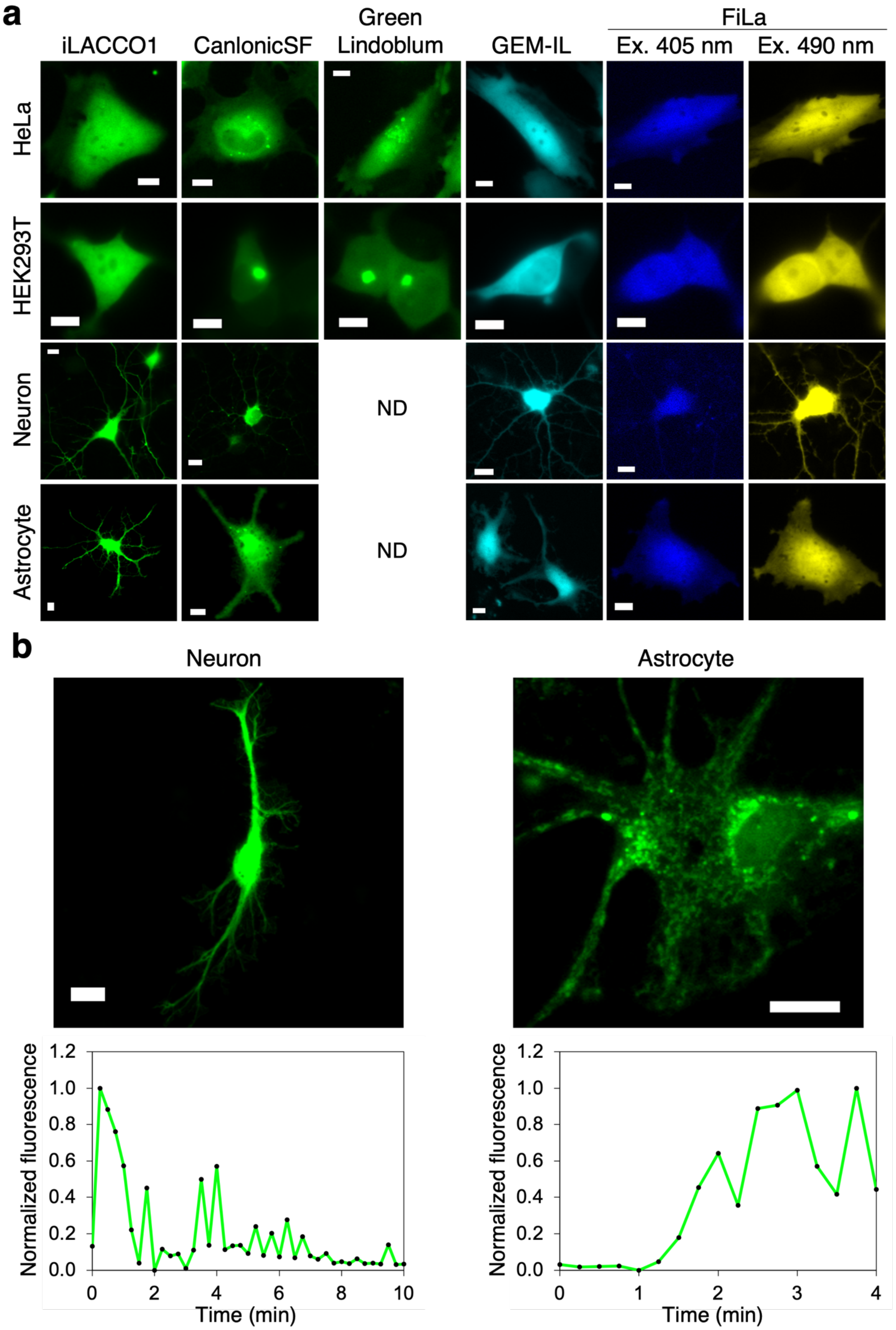
a
iLACCO1
CanlonicSF
Green Lindoblum
GEM-IL
FiLa
Ex. 405 nm
Ex. 490 nm
HeLa
HEK293T
Neuron
Astrocyte
ND
ND
b
Neuron
Astrocyte
Normalized fluorescence
Time (min)
1.2
1.0
0.8
0.6
0.4
0.2
0.0
0
2
4
6
8
10
1
3

Supplementary Figure 12. Fluorescence imaging of the other currently-available intracellular L-lactate biosensors.

(**a**) Representative images of previously-reported biosensors for intracellular L-lactate. Similar results were observed in more than 10 cells. CanlonicSF and Green Lindoblum showed large fluorescent puncta. We did not observe the detectable fluorescence of Green Lindoblum in primary cultured neurons, astrocytes, and visual cortex neurons *in vivo*. ND, not detected. Scale bars, 10 μm. (**b**) Fluorescence oscillation in a CanlonicSF-expressing primary neuron and astrocyte. Similar to eLACCO biosensors, CanlonicSF is based on TTHA0766 L-lactate binding protein that depends on Ca^{2+} concentration. These fluorescence oscillations of CanlonicSF-expressing cells are presumably due to Ca^{2+} oscillation by spontaneous neural activity. Scale bars, 20 μm.

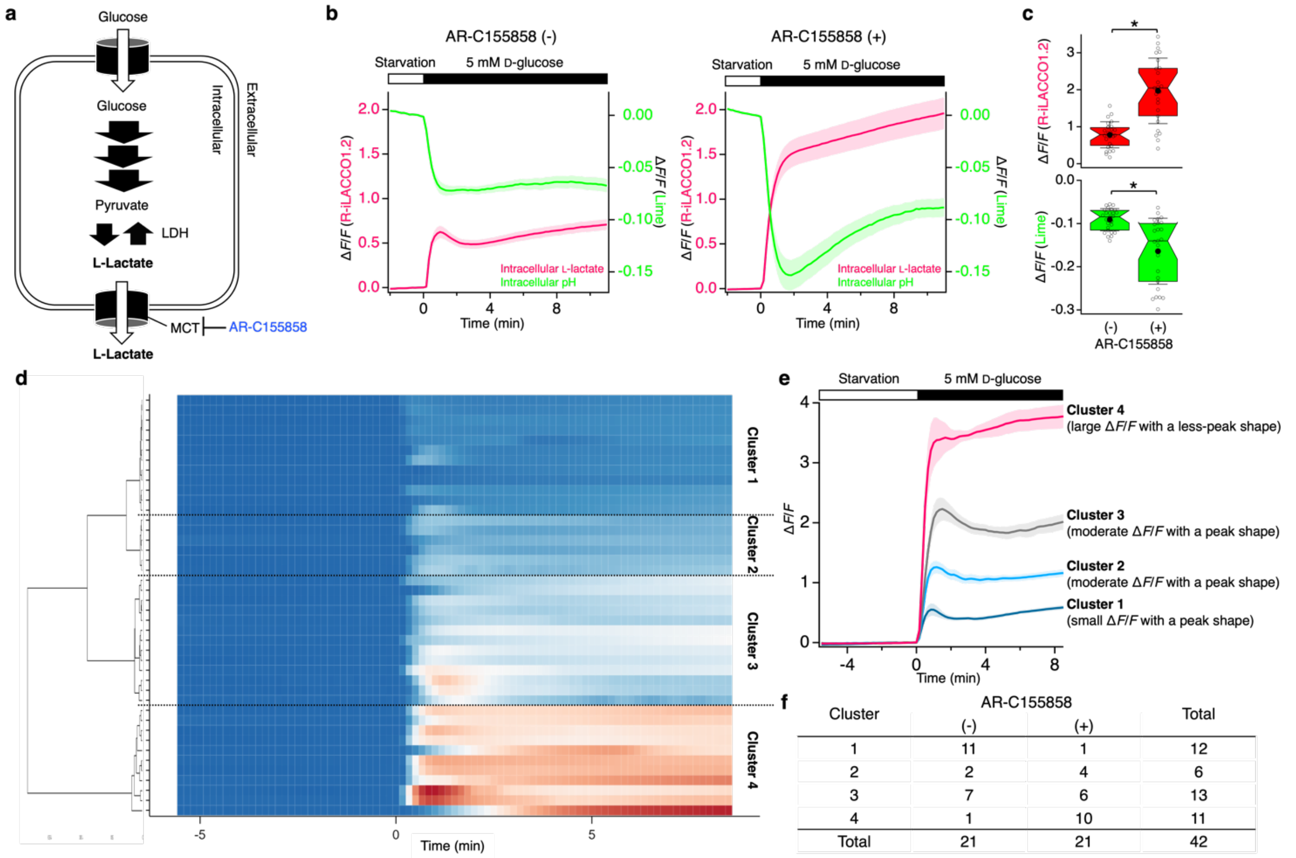

Cluster	AR-C155858 (-)	AR-C155858 (+)	Total
1	11	1	12
2	2	4	6
3	7	6	13
4	1	10	11
Total	21	21	42

Supplementary Figure 13. Inhibition of L-lactate transporter perturbs intracellular L-lactate and pH dynamics.

(**a**) Schematic representation of L-lactate metabolism. AR-C155858 acts on MCT to inhibit L-lactate transport across cell membranes. MCT monocarboxylate transporter, LDH lactate dehydrogenase. (**b**) Fluorescence traces of R-iLACCO1.2 and pH biosensor Lime expressed in starved T98 cells upon glucose treatment in the presence (right) and absence (left) of 1 μM AR-C155858. Mean ± s.e.m. Lime is an improved variant of superecliptic pHluorin[4,5]. (**c**) Maximum Δ*F/F* of R-iLACCO1.2 and minimum Δ*F/F* of Lime in glucose-stimulated T98G cells. For (b) and (c), n = 26 (R-iLACCO1.2, AR-C155858 (-)), 26 (Lime, AR-C155858 (-)), 25 (R-iLACCO1.2, AR-C155858 (+)), and 25 cells (Lime, AR-C155858 (+)). Two-tailed Student's *t*-test. *p < 0.0001 (**d**) Heatmap representation of Δ*F/F* of R-iLACCO1.2 in the presence and absence of 1 μM AR-C155858 in glucose-stimulated T98G cells. Fluorescence response patterns were hierarchically clustered by Δ*F/F* using Ward's method and separated into four clusters based on the dendrogram. (**e**) Fluorescence trace of each cluster in the glucose-treated T98G cells. n = 12, 6, 13, and 11 cells for cluster 1, 2, 3, and 4, respectively. Mean ± s.e.m. (**f**) Summary of the number of the T98G cells in each cluster.

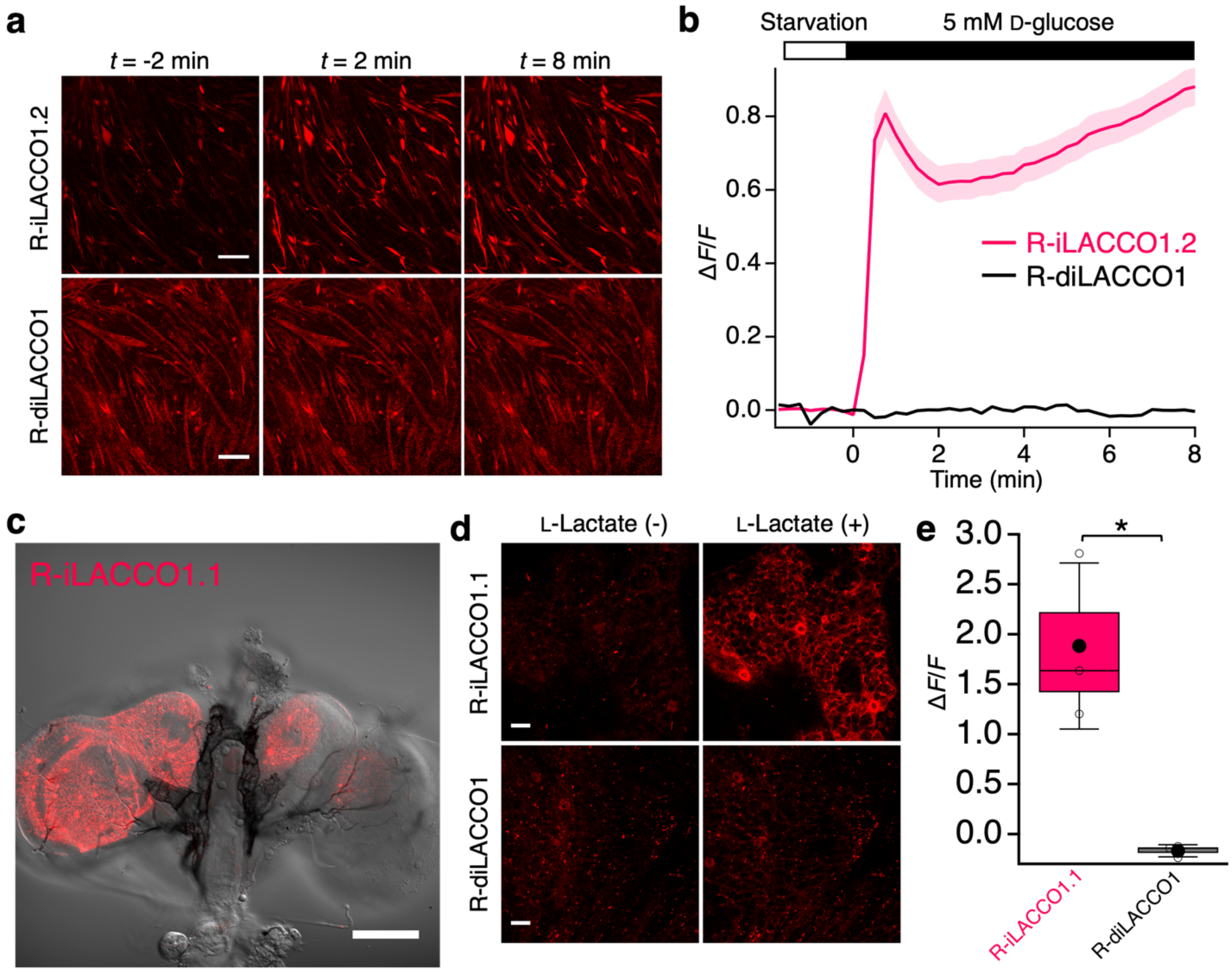

Supplementary Figure 14. Imaging of R-iLACCO variants in various tissues.

(**a**) Representative images of R-iLACCO1.2 or R-diLACCO1 expressed in differentiated C2C12 myotubes before and after 5 mM glucose treatment at *t* = 0 min. Similar results were observed in more than 10 cells. Scale bars, 200 μm. (**b**) Fluorescence traces of R-iLACCO1.2 and R-diLACCO1 in the myotubes upon glucose treatment. *n* = 108 and 81 cells for R-iLACCO1.2 and R-diLACCO1, respectively. Mean ± s.e.m. (**c**) Adult brain of the transgenic *Drosophila* expressing R-iLACCO1.1. R-iLACCO1.1 is expressed in surface glial cells. Scale bar, 100 μm. (**d**) *Ex vivo* imaging of R-iLACCO1.1 and R-diLACCO1. *Drosophila* adult brains in HL3 buffer (70 mM NaCl, 5 mM KCl, 20 mM $MgCl_2$, 10 mM $NaHCO_3$, 115 mM sucrose, 5 mM HEPES; pH 7.2) containing 5 mM glucose, 1 mM L-lactate and 0.5 mM pyruvate were exposed to 6 mM oxamate, followed by treatment with 10 mM L-lactate[6]. Scale bars, 10 μm. (**e**) *ΔF/F* of R-iLACCO1.1 and R-diLACCO1 expressed in the transgenic *Drosophila* adult brains upon treatment with 10 mM L-lactate. *n* > 100 cells from 3 brains. The horizontal line is the median; the top and bottom horizontal lines are the 25th and 75th percentiles for the data; and the whiskers extend one standard deviation range from the mean represented as black filled circle. Two-tailed Student's *t*-test. *p = 0.0131

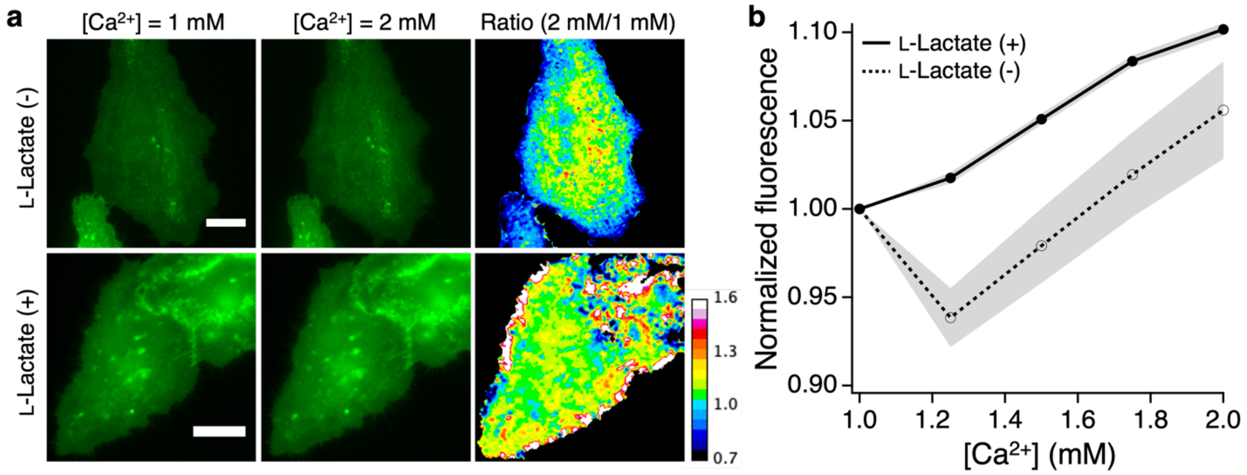

Supplementary Figure 15. Fluorescence imaging of eLACCO2.1 in extracellular Ca^{2+} concentration range from 1 to 2 mM.

(**a**) Fluorescence images of eLACCO2.1 expressed on HeLa cells in the presence (10 mM) and absence of L-lactate. Scale bars, 20 µm. (**b**) Fluorescence traces of eLACCO2.1 on HeLa cells in the presence (10 mM) and absence of L-lactate. *n* = 14 and 9 cells for L-lactate (+) and L-lactate (-), respectively. Mean ± s.e.m.

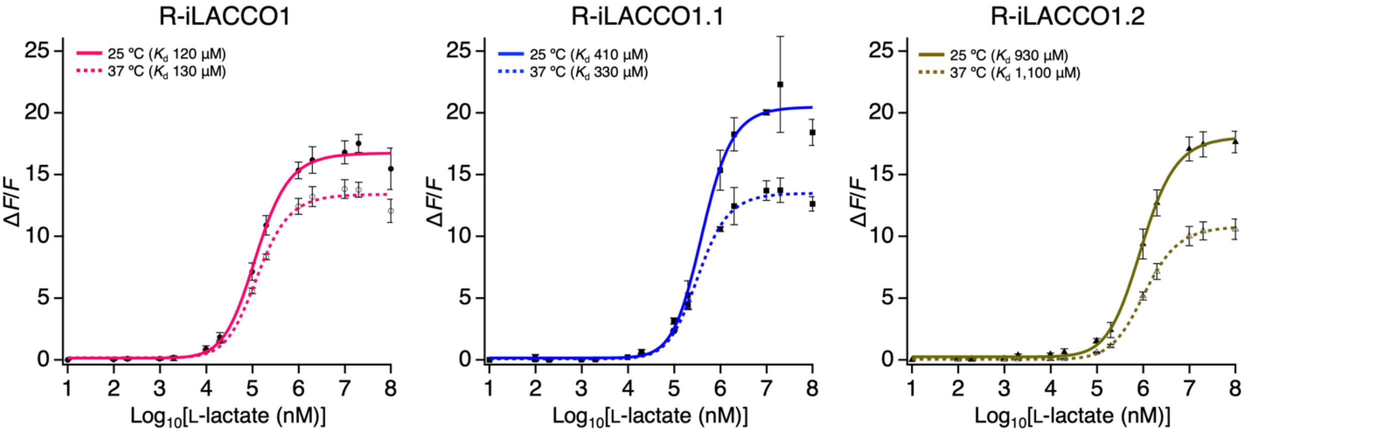

Supplementary Figure 16. Temperature-dependent lactate response of R-iLACCO variants.

Dose-response curves of R-iLACCO1 and its affinity variants for L-lactate at 25 °C and 37 °C. *n* = 3 experimental triplicates (mean ± s.d.).

Supplementary Table 1. Biochemical parameters of eLACCO variants.

	eLACCO2.1		deLACCO1		eLACCO1.1[a]		iLACCO1[b]		jGCaMP8s[c]	
L-Lactate	-	+	-	+	-	+	-	+	-[d]	+[d]
In vitro (purified protein)										
Fluorescence excitation maximum (nm)	492	495	ND	ND	493	493	492	492	N/A	N/A
Fluorescence emission maximum (nm)	509	509	512	512	510	510	510	510	N/A	N/A
pK_a	9.0	6.8	6.9	7.0	9.4	7.1	8.8	7.4	7.7	6.5
$\Delta F/F$	14[e]		0.0[e]		4[e]		30[e]		50[f]	
K_d (L-lactate) (mM)	0.96		N/A		3.9		0.36		N/A	
Hill coefficient (L-lactate)	0.98		N/A		0.94		0.6		N/A	
K_d (Ca^{2+}) (μM)	25		N/A		2.0		N/A		0.046	
Hill coefficient (Ca^{2+})	1.0		N/A		1.4		N/A		2.2	
In situ (in live mammalian cultured cells)										
$\Delta F/F$ (HeLa)	8.8[e]		0.1[e]		6.4[e]		12[g]		N/A	
$\Delta F/F$ (neuron)	8.1[e]		0.1[e]		1.5[e]		ND		4.1[h]	
$\Delta F/F$ (astrocyte)	7.3[e]		-0.2[e]		4.9[e]		ND		N/A	
K_d (L-lactate) (mM)	0.58		N/A		1.6		4.8		N/A	
Hill coefficient (L-lactate)	1.1		N/A		ND		ND		N/A	
K_d (Ca^{2+}) (μM)	270		N/A		360		N/A		N/A	
Hill coefficient (Ca^{2+})	1.2		N/A		1.2		N/A		N/A	

Mean ± s.d. N/A, not applicable.

a. Ref. [1].

b. Ref. [7].

c. Ref. [8].

d. Ca^{2+}.

e. 10 mM L-lactate.

f. 39 μM Ca^{2+}.

g. Δ*F*/*F* measured in HeLa cells pretreated with 500 μM iodoacetate, 10 μM nigericine, and 2 μM rotenone upon addition of 10 mM L-lactate.

h. Δ*F*/*F* measured in dissociated neurons upon 160 action potentials.

Supplementary Table 2. Biochemical parameters of R-iLACCO variants.

	R-iLACCO1		R-iLACCO1.1		R-iLACCO1.2		R-diLACCO1		R-eLACCO2[a]		R-GECO1[b]	
L-Lactate	-	+	-	+	-	+	-	+	-	+	-[c]	+[c]
In vitro (purified protein)												
Fluorescence excitation maximum (nm)	574	564	574	562	576	566	564	566	578	566	577	561
Fluorescence emission maximum (nm)	604	594	606	594	604	592	596	594	602	594	600	589
pK_a	9.2	5.7, 9.6	9.4	5.7, 9.7	9.2	5.7, 9.6	5.6, 9.1	5.8, 9.3	8.8	6.1	8.9	6.6
$\Delta F/F$	20[d]		22[d]		15[d]		-0.3[d]		20[d]		15[e]	
K_d (L-lactate) (μM)	74		230		350		N/A		460		N/A	
Hill coefficient (L-lactate)[i]	1.0		1.1		1.1		N/A		1.1		N/A	
K_d (Ca^{2+}) (μM)	N/A		N/A		N/A		N/A		7.6		0.48	
Hill coefficient (Ca^{2+})	N/A		N/A		N/A		N/A		1.2		2.1	
In situ (in live mammalian cultured cells)												
$\Delta F/F$ (HeLa)	5.5[f]		7.6[f]		11[f]		0.1[f]		4.5[d]		3.9[g]	
$\Delta F/F$ (neuron)	0.05[h]		0.12[h]		0.31[h]		-0.04[h]		4.3[f]		N/A	
$\Delta F/F$ (astrocyte)	0.19[h]		0.57[h]		0.49[h]		-0.03[h]		4.7[f]		N/A	
K_d (L-lactate) (mM)	0.68		3.0		4.0		N/A		0.56		N/A	
Hill coefficient (L-lactate)	1.1		1.2		1.1		N/A		1.1		N/A	
K_d (Ca^{2+}) (μM)	N/A		N/A		N/A		N/A		310		N/A	
Hill coefficient (Ca^{2+})	N/A		N/A		N/A		N/A		1.2		N/A	

Mean ± s.d. N/A, not applicable.

a. Ref. [2].

b. Ref. [9].

c. Ca^{2+}.

d. 10 mM L-lactate.

e. 39 μM Ca^{2+}.

f. Δ*F*/*F* measured in HeLa cells pretreated with 500 μM iodoacetate, 10 μM nigericine, and 2 μM rotenone upon addition of 10 mM L-lactate.

g. Δ*F*/*F* measured in HeLa cells upon treatment with Ca^{2+}, EGTA, and ionomycin.

h. Δ*F*/*F* measured in dissociated neurons or astrocytes upon 1 μM AR-C155858 treatment.

i. R-iLACCO1 and its affinity variants have Hill coefficients of ~1, whereas a green fluorescent biosensor iLACCO1 based on the same LldR and cpGFP displays a Hill coefficient of 0.6 (**Supplementary Table 1**). These results suggest that the L-lactate-dependent fluorescence response of iLACCO1 is negatively cooperative, but that of R-iLACCO1 variants is not cooperative. The original LldR protein exists as a dimer under physiological conditions[10]. Insertion of cpmApple into LldR may have abrogated the dimeric interaction between LldR protomers, leading to loss of cooperative interactions between the protomers. For reasons that remain unclear to us, insertion of cpGFP into the same site of LldR may have not disrupted the dimeric property in the same way that cpmApple did. Notably, the DNA binding domains of the LldR transcription factor were removed in both R-iLACCO1 and iLACCO1.

Supplementary Note 1: *In vitro* characterization of eLACCO2.1.

Addition of L-lactate changes the absorbance spectrum in a ratiometric manner, consistent with a shift in the equilibrium from a neutral form-dominant state to an anionic form-dominant state (**Table 1** and **Supplementary Fig. 6a**). eLACCO2.1 in the presence of L-lactate has an excitation maximum at 495 nm and the emission maximum is 509 nm (**Fig. 1e**). The molecular brightness of eLACCO2.1 in the L-lactate-bound state is 137% and 100% of eLACCO1.1 and EGFP, respectively (**Table 1**). The two-photon excitation maximum of L-lactate bound eLACCO2.1 is 932 nm with a brightness of F_2 = 20 GM that is 25% higher than L-lactate-bound eLACCO1.1 (**Table 1** and **Supplementary Fig. 6c**). The $\Delta F_2/F_2$ value in the 932–1000 nm wavelength ranges from 20–25, which is a 2–3-fold higher than eLACCO1.1 (**Table 1** and **Supplementary Fig. 6c**).

eLACCO2.1 has an apparent K_d of 960 μM for L-lactate and a Hill coefficient of 0.98 (**Supplementary Fig. 6b**). eLACCO2.1 has an apparent K_d of 25 μM for Ca^{2+} and effectively exhibits its full response to L-lactate at Ca^{2+} concentrations greater than 520 μM (**Supplementary Fig. 6d**). eLACCO2.1 exhibits pK_a values of 6.8 and 9.0 in the presence and absence of L-lactate, respectively (**Supplementary Fig. 6e**). The control biosensor deLACCO1 has no response to L-lactate (**Supplementary Fig. 6b**), and pH dependence that is similar to the L-lactate-bound state of eLACCO2.1 (**Supplementary Fig. 4b**). eLACCO2.1 is highly specific for L-lactate over a wide array of metabolites (**Supplementary Fig. 6f**). eLACCO2.1 responds to D-lactate with an apparent K_d of 8.9 mM (**Supplementary Fig. 6f inset**), a concentration that is far greater than the physiological concentration (tens of μM) in plasma[11].

Supplementary Note 2: *In vitro* characterization of R-iLACCO1.

As with eLACCO2.1, R-iLACCO1 changes absorbance peaks, indicative of the neutral (protonated) and the anionic (deprotonated) chromophore, in a ratiometric manner upon L-lactate treatment (**Supplementary Fig. 10a**). The molecular brightness of R-iLACCO1 in the L-lactate bound state is 94% and 40% of the R-GECO1 in Ca^{2+} bound state and the parent mApple, respectively (**Table 2**). Under one-photon excitation conditions, R-iLACCO1 in the absence of L-lactate displays an excitation peak at 574 nm and an emission peak at 604 nm. In the presence of L-lactate, these peak positions are slightly blue-shifted to 564 nm and 594 nm, respectively (**Fig. 1h**). The absorbance peak for the anionic form undergoes a similar blue-shift from 579 nm to 561 nm (**Supplementary Fig. 10a**). The two-photon excitation spectra of R-iLACCO1 show two overlapped peaks in both the presence and absence of L-lactate (**Supplementary Fig. 10c**), similar to those of R-GECO1 (ref. [12]). R-iLACCO1 in the absence of L-lactate displays peaks at 1080 nm and 1152 nm, while it exhibits peaks at 1048 nm and 1116 nm in the presence of L-lactate (**Supplementary Fig. 10c**). This result indicates that R-iLACCO1 blue shifts upon L-lactate binding under two-photon excitation, consistent with behavior under one-photon excitation. The two-photon brightness of L-lactate-bound R-iLACCO1 excited at 1048 nm, F_2 = 9.2 GM, is larger than that of Ca^{2+}-bound R-GECO1 (F_2 = 5 GM) (**Table 2**). Similar to some red fluorescent genetically encoded biosensors[12], the L-lactate induced two-photon excited fluorescence change ($\Delta F_2/F_2$ = 33 at 1048 nm) is larger than the one-photon excited fluorescence change ($\Delta F/F$ = 20 at 593 nm) (**Fig. 1h** and **Supplementary Fig. 10c**).

R-iLACCO1, R-iLACCO1.1, and R-iLACCO1.2 display apparent K_ds of 74 μM, 250 μM, and 350 μM for L-lactate, respectively (**Supplementary Fig. 10b**). As it incorporates the LldR L-lactate binding domain, R-iLACCO1's function is independent of Ca^{2+} concentration (**Supplementary Fig. 10d**). R-iLACCO1 has a biphasic pH dependence (pK_a values of 5.7 and 9.6) in the presence of L-lactate, and a monophasic pH dependence (pK_a value of 9.2) in the absence of L-lactate (**Supplementary Fig. 10e**). R-diLACCO1 showed no response to L-lactate (**Supplementary Fig. 10b**), and pH dependence that was similar to the L-lactate-bound state of R-iLACCO1 (**Supplementary Fig. 4d**). R-iLACCO1 is highly specific for L-lactate over other metabolites (**Supplementary Fig. 10f**), but does exhibit a substantial fluorescence response to D-lactate with an apparent K_d of 2.1 mM (**Supplementary Fig. 10f inset**).

Supplementary Note 3: Importance of leader and anchor optimization for extracellular biosensors

When targeted to the membrane of cultured neurons using a CD59-derived leader and anchor domains (CD59-eLACCO1.1-CD59), the previously reported eLACCO1.1 exhibited fluorescence puncta that were suggestive of biosensor aggregation[1]. Previous studies suggest that the specific choice of N-terminal leader sequence and C-terminal anchor domain can play an important role in the efficiency of membrane localization of biosensors on the cell surface[1,2,13]. To achieve improved membrane localization with less puncta, we screened various combinations of leader and anchor domain and finally identified HA and NGR as the best leader and anchor domain, respectively. This screening revealed that the efficiency of membrane localization exhibited a stronger dependence on the anchor domains than on the leader sequence and the leader sequences seemed to have an impact on the expression level (**Fig. 2b–e**). In this study we separately screened the anchor domains first followed by the leader sequences second. It is possible that screening of a combinatorial library of leader and anchor might provide another optimal combination. The best leader/anchor combination of the green fluorescent glutamate biosensor iGluSnFR3 (ref. [13]) has been identified as Igκ (immunoglobulin κ chain derived from *Mus musculus*) and NGR, respectively, based on the neuron-based screening similar to this study. In other work, we have recently identified Igκ and COBRA (GPI anchor derived from *Arabidopsis thaliana*) as the best leader/anchor combination for the red fluorescent extracellular L-lactate biosensor R-eLACCO2 (ref. [2]) based on screening with HEK293 and HeLa cells. These studies suggest that the best leader/anchor combination depends on the specific biosensor and the screening method. Notably, consistent with other recent works[1,2,13], the present study suggests that lipid-based GPI anchors generally provide better biosensor performance than peptide-based anchors such as a transmembrane domain of PDGFR (platelet-derived growth factor receptor derived from *Homo sapiens*), which is widely used for protein expression on cell surface. The optimized HA-eLACCO1.1-NGR expression construct has greatly improved efficiency of membrane localization, without fluorescent puncta, and expression levels compared to CD59-eLACCO1.1-CD59. These optimized properties were retained when eLACCO1.1 was switched to eLACCO2.1, in terms of membrane localization, $\Delta F/F$, and kinetics.

Supplementary References

1. Nasu, Y. *et al.* A genetically encoded fluorescent biosensor for extracellular L-lactate. *Nat. Commun.* **12**, 7058 (2021).
2. Nasu, Y. *et al.* A red fluorescent genetically encoded biosensor for extracellular L-lactate. *bioRxiv* 2022.08.30.505811 (2022) doi:10.1101/2022.08.30.505811.
3. Nasu, Y., Shen, Y., Kramer, L. & Campbell, R. E. Structure- and mechanism-guided design of single fluorescent protein-based biosensors. *Nat. Chem. Biol.* **17**, 509–518 (2021).
4. Sankaranarayanan, S., De Angelis, D., Rothman, J. E. & Ryan, T. A. The use of pHluorins for optical measurements of presynaptic activity. *Biophys. J.* **79**, 2199–2208 (2000).
5. Shen, Y. *et al.* Rational engineering of an improved genetically encoded pH sensor based on superecliptic pHluorin. *ACS Sens.* **8**, 3014–3022 (2023).
6. Aburto, C. *et al.* Single-Fluorophore Indicator to Explore Cellular and Sub-cellular Lactate Dynamics. *ACS Sens* **7**, 3278–3286 (2022).
7. Le, G. N. T. *et al.* High performance genetically-encoded green fluorescent biosensors for intracellular L-lactate. *bioRxiv* 2022.10.19.512892 (2022) doi:10.1101/2022.10.19.512892.
8. Zhang, Y. *et al.* Fast and sensitive GCaMP calcium indicators for imaging neural populations. *Nature* **615**, 884–891 (2023).
9. Zhao, Y. *et al.* An expanded palette of genetically encoded Ca^{2+} indicators. *Science* **333**, 1888–1891 (2011).
10. Gao, Y.-G. *et al.* Structural and functional characterization of the LldR from Corynebacterium glutamicum: a transcriptional repressor involved in L-lactate and sugar utilization. *Nucleic Acids Res.* **36**, 7110–7123 (2008).
11. Brandt, R. B., Siegel, S. A., Waters, M. G. & Bloch, M. H. Spectrophotometric assay for D-(-)-lactate in plasma. *Anal. Biochem.* **102**, 39–46 (1980).
12. Molina, R. S. *et al.* Understanding the Fluorescence Change in Red Genetically Encoded Calcium Ion Indicators. *Biophys. J.* **116**, 1873–1886 (2019).
13. Aggarwal, A. *et al.* Glutamate indicators with improved activation kinetics and localization for imaging synaptic transmission. *Nat. Methods* **20**, 925–934 (2023).
