# Supplementary material for: Lactate biosensors for spectrally and spatially multiplexed fluorescence imaging
Source: Nat Commun. 2023 Oct 27;14:6598. doi: 10.1038/s41467-023-42230-5 (PMC10611801; doi:10.1038/s41467-023-42230-5)
Supplement: Supplementary file 3 — Reporting Summary [file 41467_2023_42230_MOESM3_ESM.pdf]

nature portfolio

Corresponding author(s): Yusuke Nasu and Robert E. Campbell

Last updated by author(s): Sep 22, 2023

Reporting Summary

Nature Portfolio wishes to improve the reproducibility of the work that we publish. This form provides structure for consistency and transparency in reporting. For further information on Nature Portfolio policies, see our Editorial Policies and the Editorial Policy Checklist.

Statistics

For all statistical analyses, confirm that the following items are present in the figure legend, table legend, main text, or Methods section.

n/a	Confirmed	
☐	☒	The exact sample size (*n*) for each experimental group/condition, given as a discrete number and unit of measurement
☐	☒	A statement on whether measurements were taken from distinct samples or whether the same sample was measured repeatedly
☐	☒	The statistical test(s) used AND whether they are one- or two-sided<br>*Only common tests should be described solely by name; describe more complex techniques in the Methods section.*
☒	☐	A description of all covariates tested
☐	☒	A description of any assumptions or corrections, such as tests of normality and adjustment for multiple comparisons
☐	☒	A full description of the statistical parameters including central tendency (e.g. means) or other basic estimates (e.g. regression coefficient) AND variation (e.g. standard deviation) or associated estimates of uncertainty (e.g. confidence intervals)
☐	☒	For null hypothesis testing, the test statistic (e.g. *F*, *t*, *r*) with confidence intervals, effect sizes, degrees of freedom and *P* value noted<br>*Give P values as exact values whenever suitable.*
☒	☐	For Bayesian analysis, information on the choice of priors and Markov chain Monte Carlo settings
☒	☐	For hierarchical and complex designs, identification of the appropriate level for tests and full reporting of outcomes
☒	☐	Estimates of effect sizes (e.g. Cohen's *d*, Pearson's *r*), indicating how they were calculated

Our web collection on statistics for biologists contains articles on many of the points above.

Software and code

Policy information about availability of computer code

Data collection	We used cellSens Dimension (version 2.3) and MatLab (2013) running open source scanning microscope control software ScanImage (version 3.81, HHMI/Janelia Research Campus) to run our microscopes and data acquisition modules.
Data analysis	Data analysis was performed by Excel (versions 16.50), ImageJ (Fiji version 1.0), Igor Pro (version 8.04) and Prism (GraphPad Software).

For manuscripts utilizing custom algorithms or software that are central to the research but not yet described in published literature, software must be made available to editors and reviewers. We strongly encourage code deposition in a community repository (e.g. GitHub). See the Nature Portfolio guidelines for submitting code & software for further information.

Data

Policy information about availability of data

All manuscripts must include a data availability statement. This statement should provide the following information, where applicable:

- Accession codes, unique identifiers, or web links for publicly available datasets
- A description of any restrictions on data availability
- For clinical datasets or third party data, please ensure that the statement adheres to our policy

The plasmids generated in this study have been deposited in Addgene (https://www.addgene.org/depositing/83199/). Source data are provided with this paper.

Research involving human participants, their data, or biological material

Policy information about studies with human participants or human data. See also policy information about sex, gender (identity/presentation), and sexual orientation and race, ethnicity and racism.

Reporting on sex and gender	N/A
Reporting on race, ethnicity, or other socially relevant groupings	N/A
Population characteristics	N/A
Recruitment	N/A
Ethics oversight	N/A

Note that full information on the approval of the study protocol must also be provided in the manuscript.

Field-specific reporting

Please select the one below that is the best fit for your research. If you are not sure, read the appropriate sections before making your selection.

☒ Life sciences ☐ Behavioural & social sciences ☐ Ecological, evolutionary & environmental sciences

For a reference copy of the document with all sections, see nature.com/documents/nr-reporting-summary-flat.pdf

Life sciences study design

All studies must disclose on these points even when the disclosure is negative.

Sample size	Sample size was estimated based on previously published studies (Nasu et al. Nat. Commun. 12, 7058 (2021), Hashizume et al. Pro. Sci. 31, e4440 (2022)) in the development of biosensors.
Data exclusions	No data were excluded.
Replication	All experiments were replicated. Replication number for each data is indicated in the legend of corresponding figures.
Randomization	Samples were randomly allocated into groups.
Blinding	Data collection and analyses in this study were not blinded to the group allocation, as in previous literatures (Nasu et al. Nat. Commun. 12, 7058 (2021), Hashizume et al. Pro. Sci. 31, e4440 (2022)).

Reporting for specific materials, systems and methods

We require information from authors about some types of materials, experimental systems and methods used in many studies. Here, indicate whether each material, system or method listed is relevant to your study. If you are not sure if a list item applies to your research, read the appropriate section before selecting a response.

Materials & experimental systems

n/a	Involved in the study	
☒	☐	Antibodies
☐	☒	Eukaryotic cell lines
☒	☐	Palaeontology and archaeology
☐	☒	Animals and other organisms
☒	☐	Clinical data
☒	☐	Dual use research of concern
☒	☐	Plants

Methods

n/a	Involved in the study	
☒	☐	ChIP-seq
☒	☐	Flow cytometry
☒	☐	MRI-based neuroimaging

Eukaryotic cell lines

Policy information about cell lines and Sex and Gender in Research

Cell line source(s)	HeLa cell line was purchased from ATCC (#CCL-2). T98G cell line was purchased from ATCC (#CRL-1690). HEK293FT cell line was sourced from Thermo Fisher Scientific (catalog #R70007). C2C12 cell was sourced from ATCC (CRL-1772).
Authentication	No authentication was performed as the cell lines come from reliable sources.
Mycoplasma contamination	All cell lines were negative for mycoplasma contamination.
Commonly misidentified lines (See ICLAC register)	No commonly misidentified cell lines were used in this study.

Animals and other research organisms

Policy information about studies involving animals; ARRIVE guidelines recommended for reporting animal research, and Sex and Gender in Research

Laboratory animals	Mouse (C57BL6/J, 7-9 weeks old), rat (SD, 12 weeks old), and Drosophila melanogaster (Repo-Gal4/UAS-(R-iLACCO1.1 or R-diLACCO1), 3-5 days old).
Wild animals	No wild animals were used in this study.
Reporting on sex	For rat, we use female since we use a pregnant rat to obtain a primary culture from pupils. For the ex vivo and in vivo imaging, we used male mice. We used female Drosophila melanogaster.
Field-collected samples	The study did not involved samples collected in the field.
Ethics oversight	For experiments performed at The University of Tokyo, all methods for animal care and use were approved by the institutional review committees of School of Science, The University of Tokyo. For experiments at HHMI Janelia Research Campus, all surgical and experimental procedures were in accordance with protocols approved by the HHMI Janelia Research Campus Institutional Animal Care and Use Committee and Institutional Biosafety Committee. For experiments at University of Copenhagen, the procedures involving animal care, surgery, in vivo imaging, and sample preparation were approved by the local research ethics committee (Department of Experimental Medicine, University of Copenhagen) and conducted in accordance with the Danish Animal Experiments Inspectorate. All animal experiments at Kagoshima University were approved by the Experimental Animal Research Committee of Kagoshima University (Approval number: MD22086) and the Gene Recombination Experiment Safety Committee of Kagoshima University (Approval numbers: 22S018), and were performed in accordance with the ARRIVE guidelines, the National Institutes of Health (NIH) guide for the care and use of laboratory animals, and the Code of Ethics of the World Medical Association for animal experiments (http://ec.europa.eu/environment/chemicals/lab_animals/legislation_en.htm). All surgeries and experimental procedures at Stony Brook University were reviewed and approved by the Stony Brook University Animal Use Committee and followed the guidelines of NIH. The Stony Brook University IACUC-monitored Division of Laboratory Animal Resources maintained all animals. For experiments performed at University of Calgary, all methods for animal care and use were approved by the University of Calgary Animal Care and Use Committee and were in accordance with the National Institutes of Health Guide for the Care and Use of Laboratory Animals.

Note that full information on the approval of the study protocol must also be provided in the manuscript.
